# Glutaraldehyde-enhanced autofluorescence as a general tool for 3D morphological imaging

**DOI:** 10.1242/bio.060428

**Published:** 2024-11-11

**Authors:** Miika Niemeläinen, Anna-Mari Haapanen-Saaristo, Leena M. Koskinen, Josef Gullmets, Emilia Peuhu, Annika Meinander, Sara Calhim, Ilkka Paatero

**Affiliations:** ^1^ Turku Bioscience Centre, University of Turku and Åbo Akademi University, Turku FI-20520, Finland; ^2^ Institute of Biomedicine, Cancer Laboratory FICAN west, University of Turku, Turku FI-20520, Finland; ^3^ Faculty of Science and Engineering, Cell Biology, Åbo Akademi University, BioCity, Turku FI-20520, Finland; ^4^ InFLAMES Research Flagship Center, Åbo Akademi University, Turku FI-20520, Finland; ^5^ Department of Biological and Environmental Science, University of Jyväskylä, Jyväskylä FI-40014, Finland

**Keywords:** 3D imaging, Autofluorescence, Developmental biology, Microscopy, Model organism, Non-model organism

## Abstract

Routine histochemical techniques are capable of producing vast amount of information from diverse sample types, but these techniques are limited in their ability to generate 3D information. Autofluorescence imaging can be used to analyse samples in 3D but it suffers from weak/low signal intensities. Here, we describe a simple chemical treatment with glutaraldehyde to enhance autofluorescence for 3D fluorescence imaging and to generate detailed morphological images on whole-mount samples. This methodology is straightforward and cost-effective to implement, suitable for a wide range of organisms and sample types. Furthermore, it can be readily integrated with standard confocal and fluorescence microscopes for analysis. This approach has the potential to facilitate the analysis of biological 3D structures and research in developmental biology, including studies on model and non-model organisms.

## Teaser

**Summary:** Imaging of biological samples in 3D enables analysis of complex biological structures. We developed a method to enhance autofluorescence with glutaraldehyde for 3D fluorescence imaging and analysis of whole-mount samples.

## INTRODUCTION

The microscale structure of samples is typically analysed by means of histological techniques. These methods, however, require time-consuming and laborious embedding and sectioning of the samples, which inherently limits the throughput and speed of the analysis. Furthermore, the sectioning of the tissue blocks inevitably results in the loss of 3D structural information. To address this challenge, several volumetric reconstruction methods have been developed to produce 3D structures from 2D tissue slices (Pichat et al., 2018). The sectioning of tissue with microtomes may create artefacts, and the reproduction of 3D structures from the tissue slices requires the availability of a comprehensive series of intact serial sections and dedicated equipment (Pichat et al., 2018). In many cases sectioning in predefined and controlled direction is also difficult, and results in suboptimal representation of tissue morphology (Hillman, 2000). This is particularly problematic for small samples such as small model organisms, embryos, organoids or small biopsies.

The use of thick and whole-mount samples, however, would allow fast and faithful reconstruction of 3D morphology of small animals, embryos, organoids and tissues. In addition, the 3D-imaging of whole-mount embryos allows free rotation and virtual slicing of the 3D image stack in any desired orientation. The 3D morphology in whole-mount samples has been analysed using transgenic organisms, immunostainings and autofluorescence of the samples (De Medeiros et al., 2016). The availability of transgenic techniques is limited to a small number of species. Furthermore, the generation of new lines and breeding of these species may require a significant investment of time and resources, particularly for those with long generation times and large body sizes (Rogers, 2016). Immunostainings are an option but the availability of antibodies is limited for many species (Forné et al., 2010) and whole-mount immunostaining protocols are rather lengthy, lasting many days (Inoue and Wittbrodt, 2011). The resolution of many label-free techniques such as magnetic resonance imaging (MRI) and computer tomography (CT) is too low for visualizing tissue structure at microscopic level (Gregg and Butcher, 2012). While autofluorescence imaging could provide a solution (Mori et al., 2012) many samples exhibit only low levels of autofluorescence (Croce and Bottiroli, 2014), which results in poor image quality or extended imaging times. Thus, increasing the intensity of autofluorescence by chemical or physical means would solve this problem.

In this paper, we present a methodology for whole-mount 3D-morphological analysis of whole-mount embryos, small animals and 3D-bioprinted cell cultures using glutaraldehyde-based autofluorescence enhancement.

## RESULTS

**Fig. 1.**
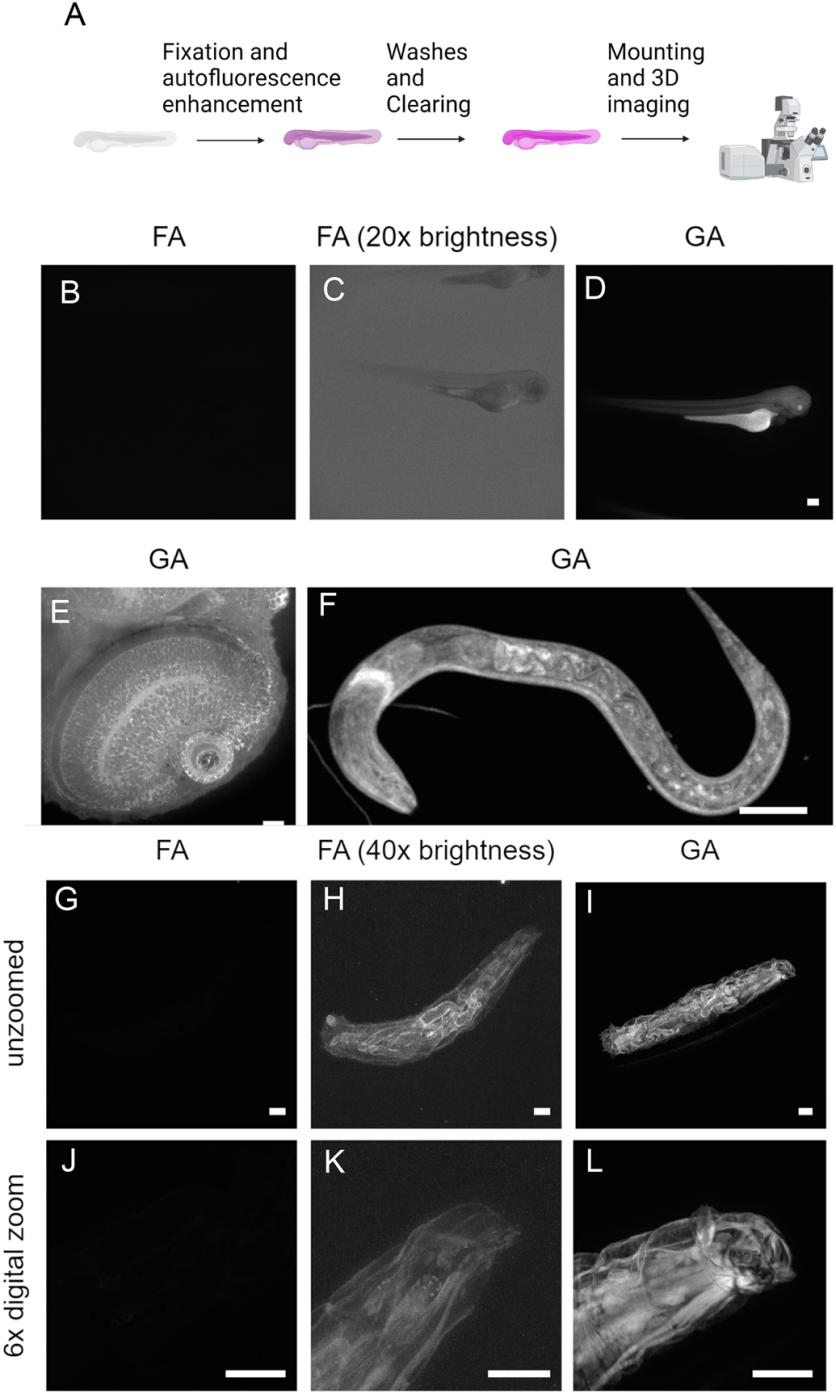
**Whole-mount 3D autofluorescence imaging of model organisms.** (A) Schematic overview of the autofluorescence enhancement process. Image created with BioRender.com. (B-D) Fluorescence microscopy image of 3 dpf whole-mount zebrafish embryos fixed with FA (B), fixed with FA and brightness digitally increased by 20× (C) or GA (D), scale bar: 200 µm. (E) Zebrafish eye imaged with high-resolution spinning disk confocal microscopy and autofluorescence enhancement. Scale bar: 20 µm. (F) Confocal image of whole-mount *C. elegans* larvae processed with GA fix. *n*=4. Scale bar: 20 µm. (G-L) Structural illumination fluorescence image of whole-mount *Drosophila* larvae fixed with FA (G,J), brightness increased by 40× (H,K) or GA (I,L). *n*=4 in both groups. Maximum Z-projections in F-J. Scale bar: 200 µm.

Given that glutaraldehyde has been demonstrated to induce autofluorescence (Collins and Goldsmith, 1981), we compared the generation of the autofluorescence in 3D samples by fixing gelatin gels with formalin (FA) or glutaraldehyde (GA). The glutaraldehyde fixation yielded robust fluorescence in gelatin gels across a broad concentration range compared to FA (Fig. S1A). The absorbance spectrum was wide (Fig. S1B), with maximum signals being obtained relatively slowly (Fig. S1C). The autofluorescence produced by GA was 30-fold (488 nm excitation) to >100-fold (520 nm excitation) higher than that produced by FA (Fig. S1D). The glutaraldehyde autofluorescence spectra was analysed by spectral scanning in whole-mounted fish embryos, nematodes and tardigrades. As anticipated, all glutaraldehyde-fixed samples had broad emission and excitation spectra although there were some differences between samples (Fig. S1E). To utilize this phenomenon for biological imaging, we used a simple and straight-forward workflow to enhance autofluorescence in whole-mount samples (Fig. 1A).

The GA-enhanced autofluorescence imaging protocol was initially tested with whole-mount zebrafish (*Danio rerio*) embryos, a popular model organism in developmental and cancer biology, (Fig. 1B-E; Fig. S2, Movie 1) using wide-field and spinning disk confocal imaging. At low resolution imaging, the enhancement of autofluorescence by GA treatment was evident (Fig. 1B-D). At higher resolutions, more detail could be visualized, for example, fine structures of the zebrafish eye with imaging depth of approximately 100 µm (Fig. 1E; Fig.S2 and Movie 1). To test the method with a different model organism, we imaged and visualized internal structures of nematode (*Caenorhabditis elegans*) (Fig. 1F; Fig. S3, Movies 2 and 3) using spinning disk confocal microscopy. Subsequently, another model was analysed using different imaging modality: larvae of the fruit fly (*Drosophila melanogaster*) using structured illumination microscopy and ethyl cinnamate clearing (Fig. 1G-L; Fig. S4, Movies 4 and 5). The 3D autofluorescence generated by GA-fixation enabled the imaging and 3D structural analysis of both *C. elegans* and cleared *Drosophila* larvae. These results indicate that this method is suitable for morphological analysis and phenotyping of small animals and their embryos utilizing different instruments capable of 3D fluorescence imaging.

**Fig. 2.**
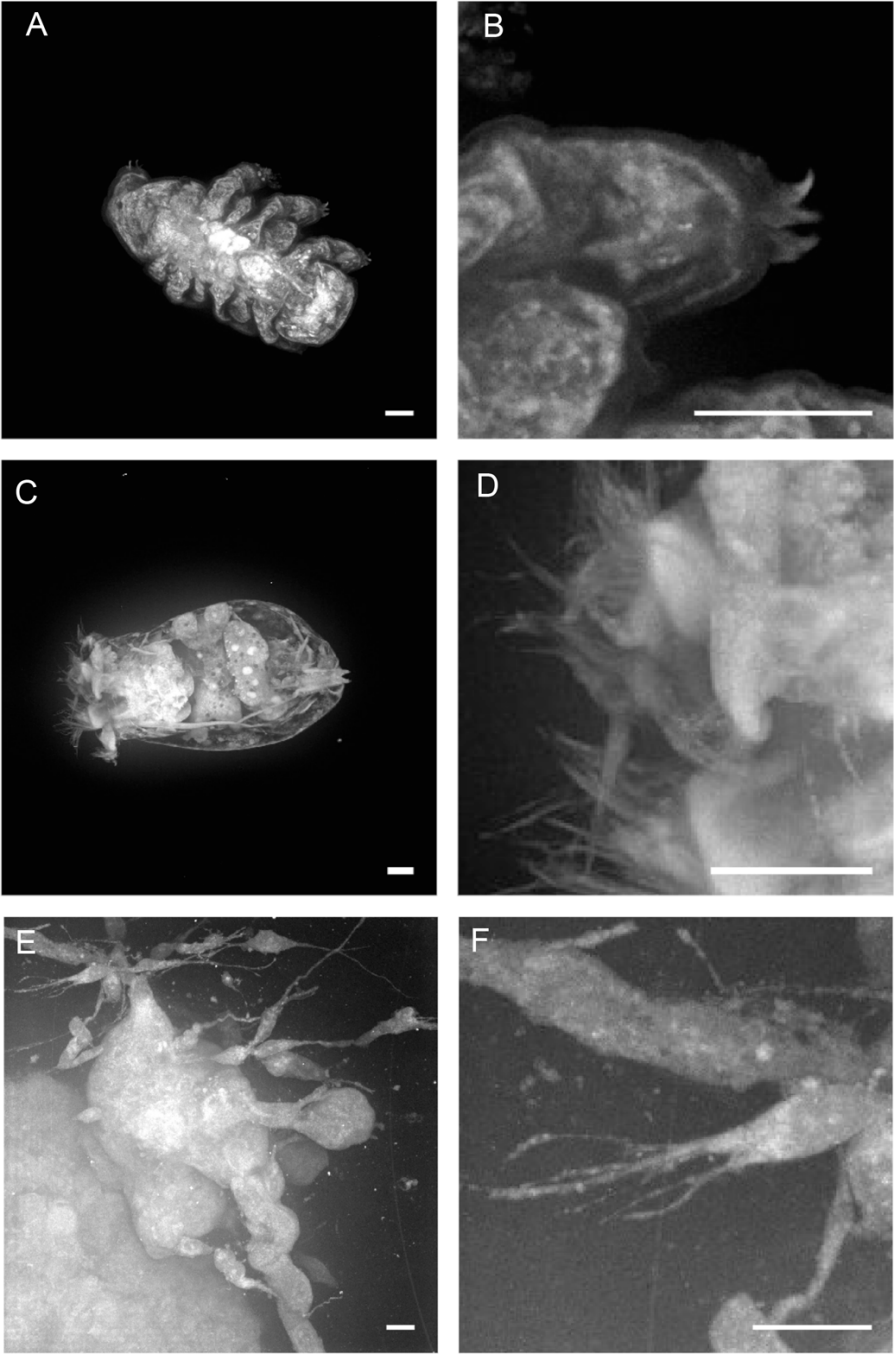
**Whole-mount 3D glutaraldehyde-enhanced autofluorescence imaging of non-model model organisms.** (A,B) Spinning disk confocal image of whole-mount tardigrade *M. ripperi* stained with FA or GA. *n*=5 tardigrades/condition. (C,D) Spinning disk confocal image of whole-mount rotifer *B. plicatilis* stained with FA or GA. *n*=4 rotifers/condition. (E,F) Spinning disk confocal images of 3D-bioprinted mammary epithelial cell cultures displaying cell invasion out from the 3D-bioprinted structure. *n*=4 bioprints. Maximum Z-projections. Scale bars: 20 µm.

When using well-established genetic model organisms with fast reproduction cycles, such as *C. elegans* or *Drosophila,* it is possible to cross animals to various transgenic reporter strains to visualize structures and phenotypes. Although many fundamental questions in cell biology would be best addressed in non-model organisms (Goldstein and King, 2016), the genetic and molecular toolbox for these organisms is comparatively limited in comparison to that available for established model organisms (Russell et al., 2017). One of the key advantages of autofluorescence imaging over immunostainings or transgenic reporter strains is that diverse samples arising from virtually any organism could be used. Indeed, the 3D autofluorescence histology was able to beautifully visualize morphology of non-model organisms such as the tardigrade *Macrobiotus ripperi* (Fig. 2A,B; Fig. S5, Movie 6) and the rotifer *Brachionus plicatilis* (Fig. 2C,D; Fig. S6, Movies 7 and 8). Consequently, our method enabled detailed morphological analysis of organisms, which lack a sophisticated and robust molecular toolbox.

In addition to model organisms, developmental biology frequently uses sophisticated *in vitro* methods including organoids and 3D-bioprinted tissues (Ollé-Vila et al., 2016). Therefore, we tested autofluorescence imaging using 3D-bioprinted mammary epithelial cell cultures (Koskinen et al., 2024) and post-fixation treatment with GA. Consequently, we conducted an experiment to assess the efficacy of autofluorescence imaging in 3D-bioprinted mammary epithelial cell cultures (Koskinen et al., 2024) with and without post-fixation treatment with GA. The GA-enhanced autofluorescence technique was indeed capable of visualizing the 3D morphology of the cultures at cellular resolution (Fig. 2E-F; Fig. S7, Movie 9), thereby indicating the applicability of this method for the analysis of complex 3D cell culture and tissue models. The 3D-bioprinted mammary epithelial cell cultures showed prominent protrusions (Fig. 2E,F). As quantitative morphological analysis is necessary in analysis of several phenotypes, we carried out quantitative analysis of these protrusions as proof-of-principle of suitability of GA-autofluorescence enhancement for quantitative morphological analysis. By using Imaris software we were able to analyse these protrusions in 3D (Fig. S8A). Interestingly, the protrusions were rather long, median length being 69 µm (Fig. S8B).

**Fig. 3.**
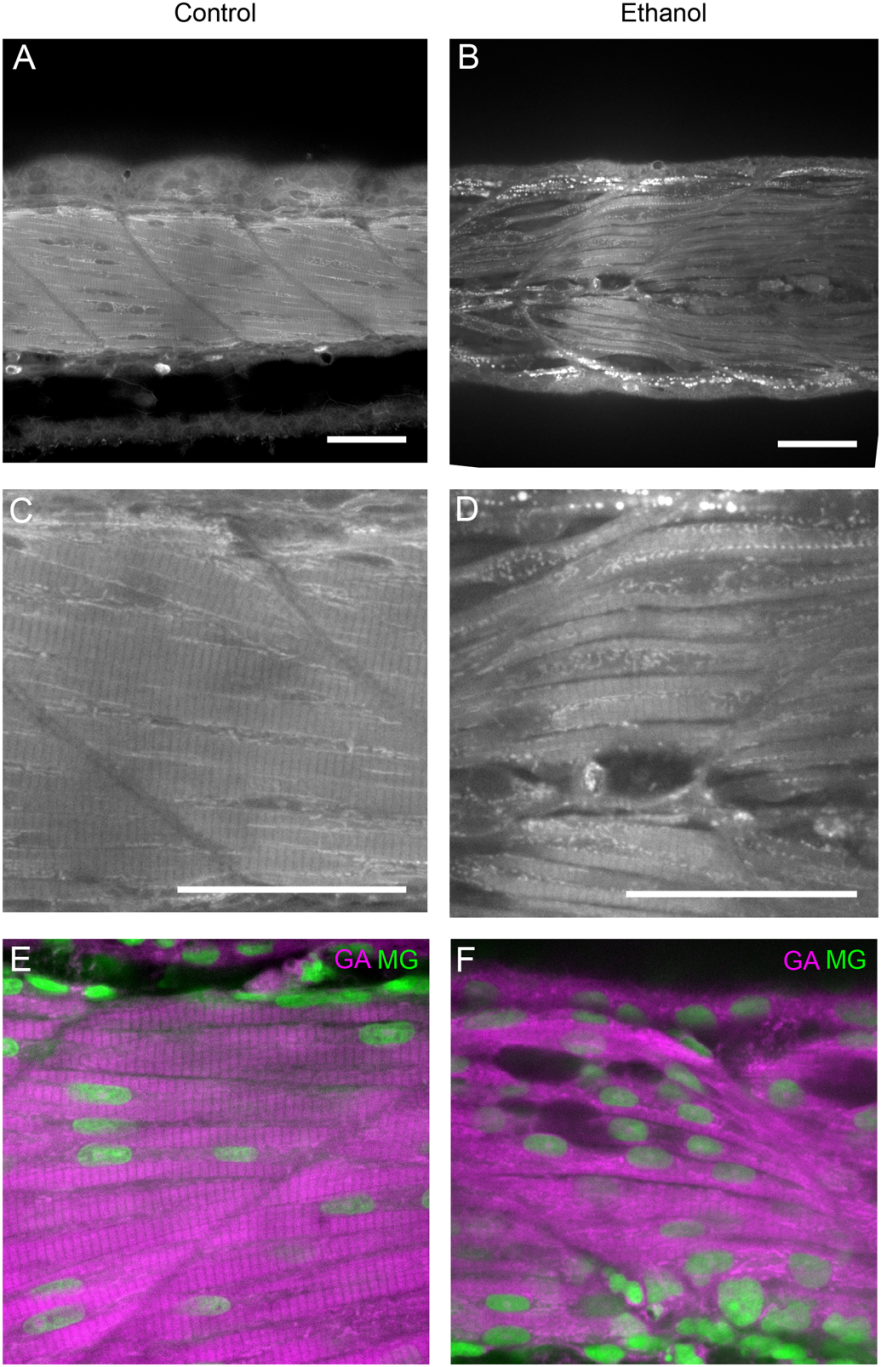
**Analysis of chemically perturbed samples using 3D imaging of glutaraldehyde-enhanced autofluorescence.** (A,B) Confocal images of 3 dpf zebrafish without (A,C,E) or with 2% ethanol (B,D,F). (A,B) overview of GA autofluorescence in zebrafish embryo musculature. (C,D) Close-up images of A and B, respectively. (E,F) Similarly treated zebrafish embryos were stained for nuclei (with Methyl Green, MG, in green) and imaged together with GA autofluorescence (GA, in magenta). Control, *n*=10; ethanol, *n*=7. Scale bars: 50 µm.

The data indicated that 3D autofluorescence imaging could represent a straightforward method for phenotypic analysis of organisms exposed to chemical compounds and mutant strains. To validate the use of autofluorescence imaging in the analysis of chemically induced phenotypes, we treated zebrafish embryos with ethanol at a sublethal concentration. Ethanol exposure has been shown to cause multi-organ damage to embryos, including damage to muscle fibres (Lovely et al., 2016; Coffey et al., 2018). Imaging of the 3D autofluorescence was able to detect ethanol-induced structural defects in muscle (Fig. 3A-D), an observation well in line with previously published (Lovely et al., 2016; Coffey et al., 2018). This suggests that the methods are suitable for analysing chemically induced defects in entire model organisms. To further test the utility of the method, we carried out co-staining with nuclear dye Methyl Green (Prieto et al., 2015), to facilitate detection of nuclei in the tissue. Indeed, when using high-resolution imaging, we were able to detect both GA autofluorescence and nuclei in the control and ethanol-treated embryos (Fig. 3E,F). Despite the broad spectrum of GA autofluorescence generating some background, the stained nuclei were clearly identifiable, providing additional information for morphological analysis.

**Fig. 4.**
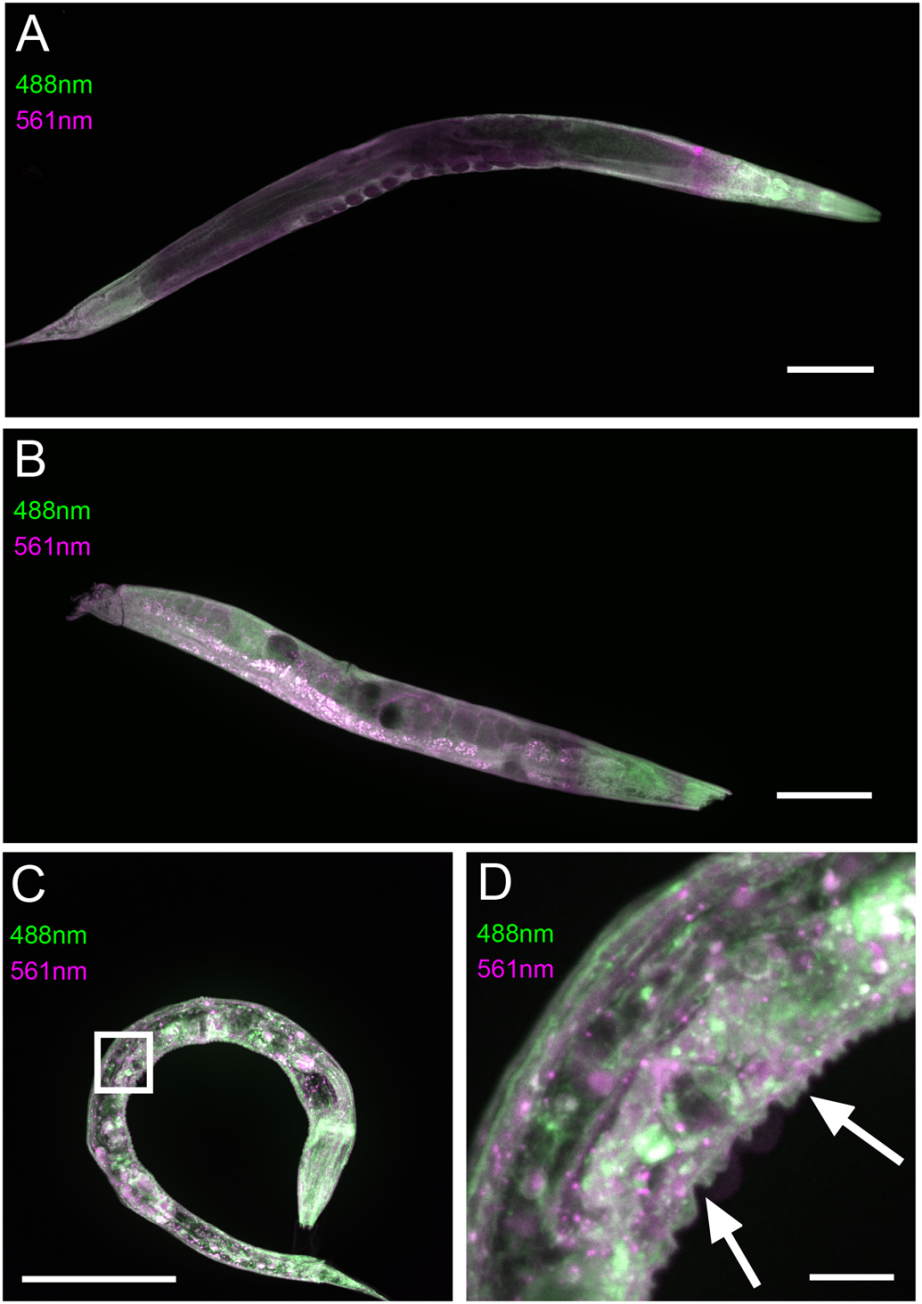
**Analysis of mutant nematodes using 3D imaging of GA-enhanced autofluorescence.** The *C. elegans* nematodes were fixed with GA and imaged with 488 nm (in green) and 561 nm (in magenta) excitation. (A) Analysis of wild-type (N2) animals using 3D autofluorescence. (B) *dpy-10* mutants have shortened and wider body form. (C,D) *bli-1* mutants have defects in cuticular morphology. (D) Zoomed image of region of interest. Cuticular blisters annotated with arrows. Maximum Z-projections. Image intensity and contrast linearly adjusted separately for each image for best visualization of morphology. *n*=4 per genotype. Scale bars: 100 µm (A,B,C); 10 µm (D).

To further validate the use of 3D autofluorescence imaging in the analysis of mutant phenotypes, we analysed homozygous viable *C. elegans dpy-10* and *bli-1* mutants. *Dpy-10* mutant exhibits shortened (dumpy) phenotype and *bli-1* mutant a blistered cuticle both due to mutations in collagen genes (Myllyharju and Kivirikko, 2004). As expected, the 3D autofluorescence imaging technique was capable of visualising altered body shape and tissue architecture of the mutants (Fig. 4A-D). Two different channels were employed for the imaging of GA-enhanced autofluorescence: excitation with 488 nm or 561 nm lasers (Fig. 4A-D). While the overall signal patterns were quite similar, there were also some structures that have differential GA-enhanced autofluorescence in the two channels (Fig. 4D), which may be useful property in analysis of various sample types.

## DISCUSSION

We envision that the presented methodology for 3D imaging using GA-enhanced autofluorescence enables new possibilities to analyse the 3D morphology of various organisms and samples. The GA-enhanced autofluorescence has previously been employed in analysis of isolated red blood cells (Alessandro et al., 2019) and plant tissues (Fester et al., 2008). However, it has not been utilized for whole-mount analysis of whole animals, tissues and embryos. The methodology can be employed using standard confocal, spinning disk, light-sheet, widefield or multiphoton microscopes, which are widely available in research institutions. Consequently, it can be rapidly applied in research projects in various fields of biology and biomedicine. The method does not necessitate physical sectioning, thereby enabling its utilisation with small and fragile samples. With larger and/or less-transparent model, optical tissue clearing will facilitate imaging deeper into the structures (Vieites-Prado and Renier, 2021) and even large samples as whole mice (Cai et al., 2023). Here, GA-enhanced autofluorescence showed compatibility with ethyl cinnamate cleared *Drosophila* larvae but as glutaraldehyde based autofluorescence is based on the stable chemical cross-links (Lee et al., 2013) it is likely that the method is compatible with most – if not all – clearing protocols. A somewhat similar approach for 3D fluorescence imaging has been recently utilized by generation of fluorescent reaction products of citrate for 3D imaging of tissues (Pac et al., 2022). Although, GA-enhanced autofluorescence has broad emission and excitation spectrum, it has been possible to combine it with specific fluorescent immunostainings (Kwan et al., 2022), multiphoton imaging (Fester et al., 2008) and potentially also with transgenic fluorescent protein reporters in model organisms. We used GA-autofluorescence method successfully with methyl green nuclear staining. However, when attempting to utilise GA with EGFP and DAPI, a high background was observed (data not shown). This phenomenon can at least partially be explained by overlapping excitation and emission spectra and unique fluorescence properties of GA (Collins and Goldsmith, 1981). The use of GA-enhanced autofluorescence with some fluorescent stains and proteins may, therefore, require more advanced imaging modalities such as fluorescence life-time imaging (Steinkamp et al., 1999) to differentiate weaker signals from the strong overlapping GA autofluorescence signal. The organisms may also have differences in the GA-autofluorescence spectrum depending on the chemical composition of the organism and its tissues. The quantitative analysis of tissue morphology based solely on fluorescence intensity-based segmentation may prove challenging due to the presence of autofluorescence in all tissues. The application of AI/ML-based segmentation training could facilitate the identification of desired cell types. This approach has been demonstrated to be effective in the segmentation of electron microscopy data (Treder et al., 2022), which exhibits monochromatic staining patterns that are somewhat analogous to the GA-enhanced autofluorescence patterns. As proof-of-principle experiment of suitability of GA-enhanced autofluorescence for quantitative morphological analyses in 3D, we successfully carried out analysis of protrusions in 3D-bioprinted mammary epithelial cell cultures. This indicates that the method is suitable not only for qualitative analyses but also for quantitative analyses of morphology.

The GA-enhanced autofluorescence method was robust as it worked with wide range GA concentration and incubation times, pre-fixed samples and multiple organisms. This indicates that the GA autofluorescence enhancement is robust enough to be used in field studies and in other challenging or low-resource environments.

Taken together, this high-resolution 3D GA-enhanced autofluorescence imaging method has potential to create significant advances in the understanding the diversity of tissue architecture during animal development, and to inaugurate new avenues in 3D morphological analysis of both model and non-model species.

## MATERIALS AND METHODS

### Reagents

All chemical reagents were purchased from Sigma-Aldrich.

### Gelatin gels and spectrophotometric analyses

Gelatin gels were prepared from porcine skin gelatin by hydrating gelatin powder in water (10% w/v) and heating in microwave oven. Once dissolved, the solution was pipetted into 96-well plate and allowed to solidify in fridge. Formed gels were fixed for various times and concentrations of formaldehyde (FA) or GA. Fluorescence imaging of gelatin gels was carried out with Azure Sapphire Biomolecular RGBNIR Imager. Absorption spectrum was measured with Shimadzu UV-1900i spectrophotometer from 350 nm to 800 nm. Kinetic absorbance measurement was done using Multiskan plate reader with 450 nm absorption.

### Processing and imaging of *D. rerio* embryos

Zebrafish embryos were obtained via natural spawning using standard procedures under a licence MMM/465/712-93 issued by Finnish Ministry of Agriculture and Forestry. Zebrafish line *casper (mitfa-/-, nacre-/-)* was used (White et al., 2008). Embryos were cultured in E3+PTU medium in 28.5°C incubator. The embryos were fixed with 4%FA/PBSTw (PBS+0.1% Tween-20) or 2.5% GA /PBSTw for overnight at RT. After fixation, the embryos were washed with PBSTw. After three washes with PBSTw, the embryos were mounted into low-melting point agarose (0.7%) on glass-bottomed dishes for imaging. For ethanol exposure, 2% of ethanol was added to the E3-medium and embryos cultured at 28.5°C for 2 days. Methyl Green stain was prepared from a 2% stock solution, diluted 1:100 in E3 media. Embryos were incubated for 30 min in either an Eppendorf or a small Petri dish and washed a few times to remove the excess dye. Low-resolution images were taken with Zeiss Axiozoom fluorescence stereomicroscope and high-resolution images with 3i spinning disk.

### Processing and imaging of *D. melanogaster* larvae

*D. melanogaster* were maintained at 25°C with a 12 h light–dark cycle on medium containing agar 0.6% (w/v), malt 6.5% (w/v), semolina 3.2% (w/v), baker's yeast 1.8% (w/v), nipagin 2.4%, and propionic acid 0.7%. W1118 *Drosophila* larvae of mixed stages were transferred to fixative (4% FA in PBSTw or 2.5% GA in PBSTw) for fixation at room temperature for at least 24 h. The larvae were stored in the fixative at RT until washed three times with PBSTw, dehydrated in isopropanol, and cleared with ethyl cinnamate as described earlier (Masselink et al., 2019). Larvae were imaged in ethyl cinnamate using AxioZoom microscope (Zeiss) with Apotome structural illumination module.

### Processing of *C. elegans*

Strains were provided by the CGC, which is funded by NIH Office of Research Infrastructure Programs (P40 OD010440). Mixed stage cultures of *C. elegans* [N2 (wild-type), CB4856 (*C. elegans* wild isolate), CB769 (bli-1(e769) II) and CB128 (dpy-10(e128) II] were maintained using standard protocols (Stiernagle, 2006). For fixation and staining, the worms were washed off from NGM plates. The worms were fixed with 2.5% glutaraldehyde in PBS for overnight at RT. After several washes with PBSTw, the worms were mounted into low-melting point agarose (0.7%) on glass-bottom dishes for imaging with 3i CSU-W1 spinning disk, 40× Zeiss LD C-Apochromat WI or 40× Zeiss LD Plan-Neofluar objective and excitation at 561 nm.

### Processing of *B. plicatilis*

Salt water rotifers (*B. plicatilis,* obtained from Planktovie, Marseille, France) were grown in 15 ppt sea salt (Royal Nature Ion Balanced Pro reef Salt, Planktovie) water +28C with constant aeration and fed with RG plus feed (Planktovie). The rotifers washed with 10 ppt salt water and fixed with 2.5% GA in 10 ppt salt water for overnight at RT. After several washes with PBS, the rotifers were mounted into low-melting point agarose (0.7%) on glass-bottomed dishes for imaging with 3i CSU-W1 spinning disk, 40× Zeiss LD C-Apochromat WI objective and excitation at 561 nm.

### Processing of *M. ripperi*

Tardigrades (*M. ripperi*) were reared in the lab since 2019 and originally collected from moss on rocks at coordinates 62°22′N, 25°77′E (Jyväskylä, Finland). Tardigrades were mounted in low melting agarose (1.2% in PBSTw) in glass bottomed dish (⌀ 35 mm) and anesthetized with Tricaine (2 mg/ml). After the animals were immobile, the anaesthetic solution was removed and replaced with fixative (2.5% GA in PBSTw). Animals were held in fixative for 1-2 h at RT. After the fixative removal the agarose, dish and animals were washed several times with PBSTw and stored in PBS until imaged (to prevent the agarose from drying). Imaging was performed with 3i CSU-W1 spinning disk, 40× Zeiss LD C-Apochromat WI objective and excitation at 488 nm and 561 nm.

### Processing of 3D-printed cultures

The 3D-bioprinted mammary epithelial cell cultures were fabricated and fixed according to the published protocol (Koskinen et al., 2024). The fixed cultures were stored in PBS supplemented with penicillin-streptomycin at +4°C before further processing. Then the cultures were treated as whole-mounts with 2.5% GA in PBS at least overnight at +4°C, and kept in this solution until imaged with 3i CSU-W1 spinning disk, 20× Zeiss LD Plan-Neofluar (NA0.4) and 40× Zeiss LD Plan-Neofluar (NA0.6) objective and excitation at 561 nm.

### Confocal fluorescence microscopy

Samples were imaged with 3i Marianas CSU-W1 spinning disk (50 µm pinholes) confocal equipped with 40× NA 1.1 water immersion objective using 488 nm excitation and GFP (525/50) emission filter, or 561 nm excitation and Cy3/Alexa 568 (617/73 nm) emission filter. Methyl Green signal was imaged with 633 nm excitation and Cy5/Alexa647 (692/40 nm) emission filter. Images were captured using Hamamatsu sCMOS Orca Flash 4.0 camera. Samples were immersed in water-based buffer for imaging.

### Image processing

Images were processed using FIJI (fiji.sc) and Imaris (Bitplane) for 2D presentation and 3D rendering. The branching structures in the bioprint data were subjected to analysis using the Filament tracing tool in Imaris, Oxford Instruments (v.10.2, Bitplane, Concord, MA, USA). First, the background noise was reduced by pre-processing the data in FIJI using rolling ball background subtraction. Then, the data was imported into Imaris. Thresholds for intensity and branching angle were adjusted to ensure accurate representation of the filaments. The detection was improved by manual detection and correction of filament tracing. Once, the filament tracing was of sufficient quality, the quantitative metrics of protrusions such as length of filaments were measured.

### Spectral analysis

A spectral analysis was conducted on a Stellaris 8 Falcon confocal microscope (Leica Microsystems) which enabled the scanning of both excitation and emission wavelengths. The microscope was equipped with a White Light Laser with tunable excitation wavelengths 440–810nm operating at speed of 400 Hz. The image format was 512×512, and the bit depth was 16. Spectral detection was performed with Power HyDX2 photon-counting detectors in the spectral window of 450–830nm. The detection was based on behind method i.e. the emission is detected in a wavelength longer than the excitation wavelength. Step size through the scanning was kept 25 nm and bandwidth 30 nm. Images were acquired with HC Plan APO CS2 20×/0.8 dry objective (Leica Microsystems). Contour plots were obtained in LAS X software for both excitation and emission.

## Supplementary Material

10.1242/biolopen.060428_sup1 Supplementary information
