# Supplementary material for: Glutaraldehyde-enhanced autofluorescence as a general tool for 3D morphological imaging
Source: Biol Open. 2024 Nov 11;13(11):bio060428. doi: 10.1242/bio.060428 (PMC11583915; doi:10.1242/bio.060428)
Supplement: Supplementary information [file biolopen-13-060428-s1.pdf]

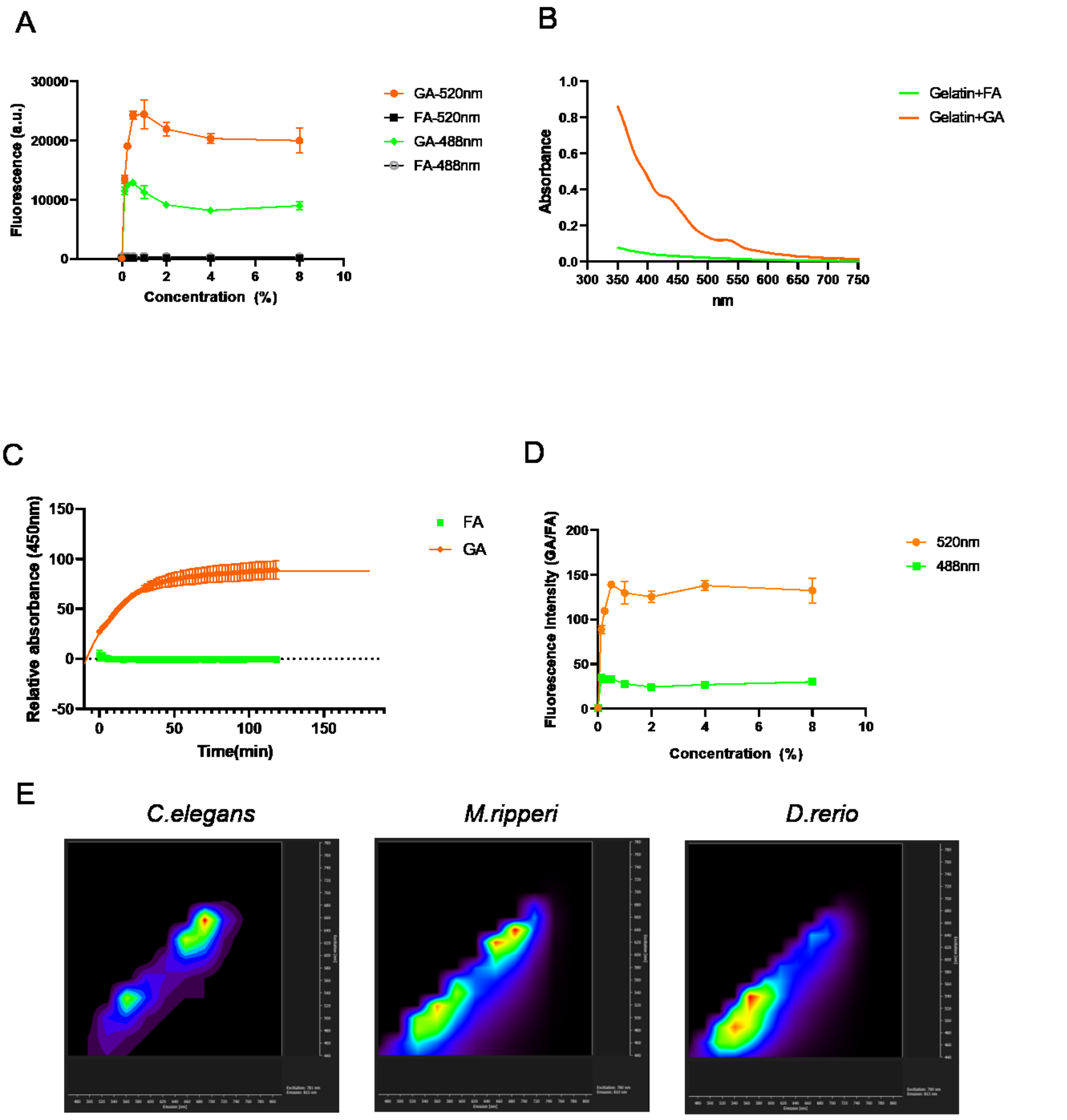

Figure S1

Fig. S1. Characterization of autofluorescence generation in vitro in gelatin gels. A) Testing of different concentrations of GA and FA. B) Absorbance spectrum of GA or FA fixed gelatin gel. C) Kinetic analysis of absorbance during FA or GA fixation. D) Relative fluorescence intensity GA vs FA in 488nm and 520nm excitation. Gelatin gels were fixed with different concentrations of FA and GA. E) Spectral analysis. White Light Laser with tunable excitation wavelengths 440–810 nm and spectral detection in window of 450–830 nm was used. Examples on spectral scans on different emission (x-axis) and excitation (y-axis) wavelengths of nematode (C.elegans, N2 strain), tardigrade (M.ripperi) and zebrafish embryo (D. rerio, strain: *mitfa, roy, tg(fli1:EGFP^{y1}).* In A, C, D, n=4 replicates and mean +/- SEM plotted. In E, two samples from each species was analysed (n=2).

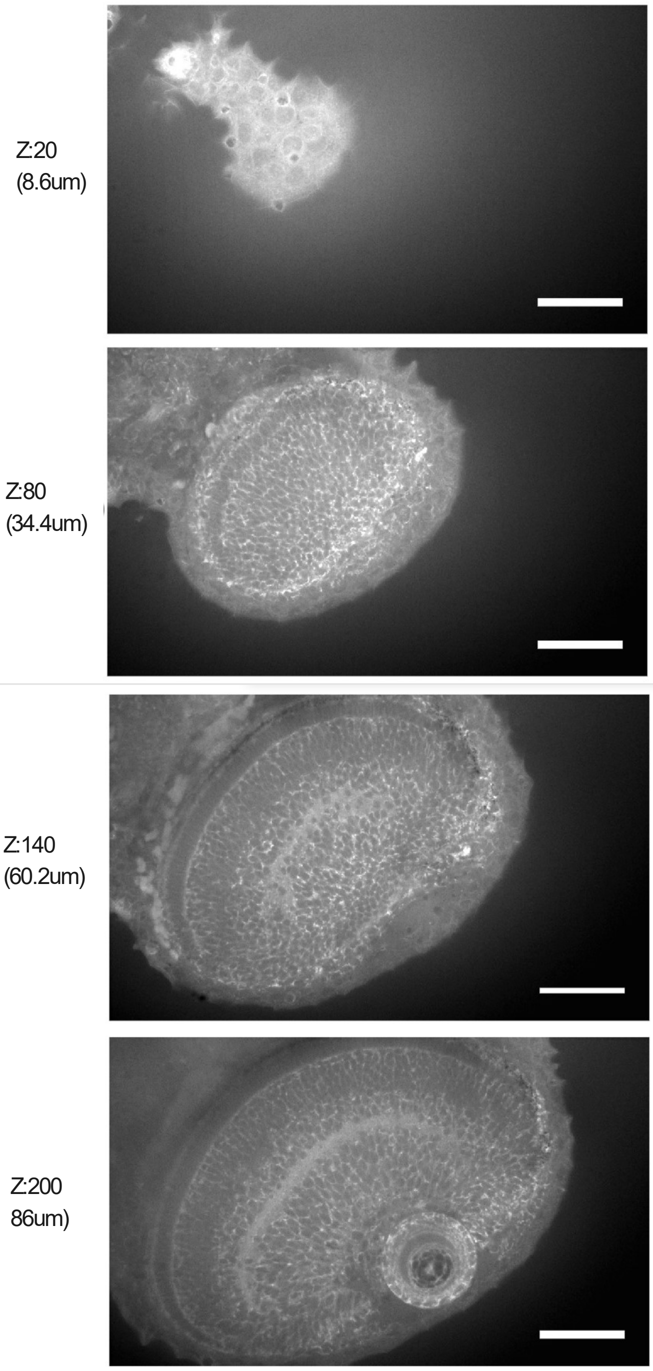

Fig. S2. Z-series of 3D autofluorescence imaging of whole-mount zebrafish embryo. Scale bar 50 µm.

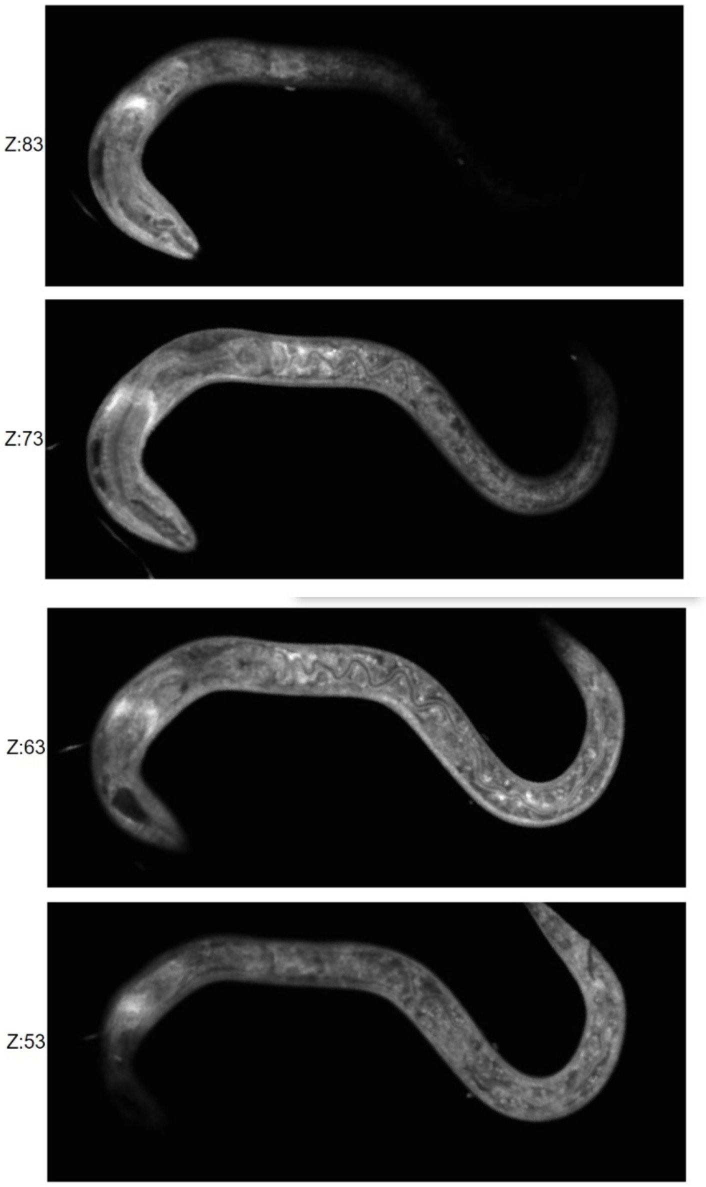

Figure S3

Fig. S3. Z-series of 3D autofluorescence imaging of *Caenorhabditis elegans* nematode.

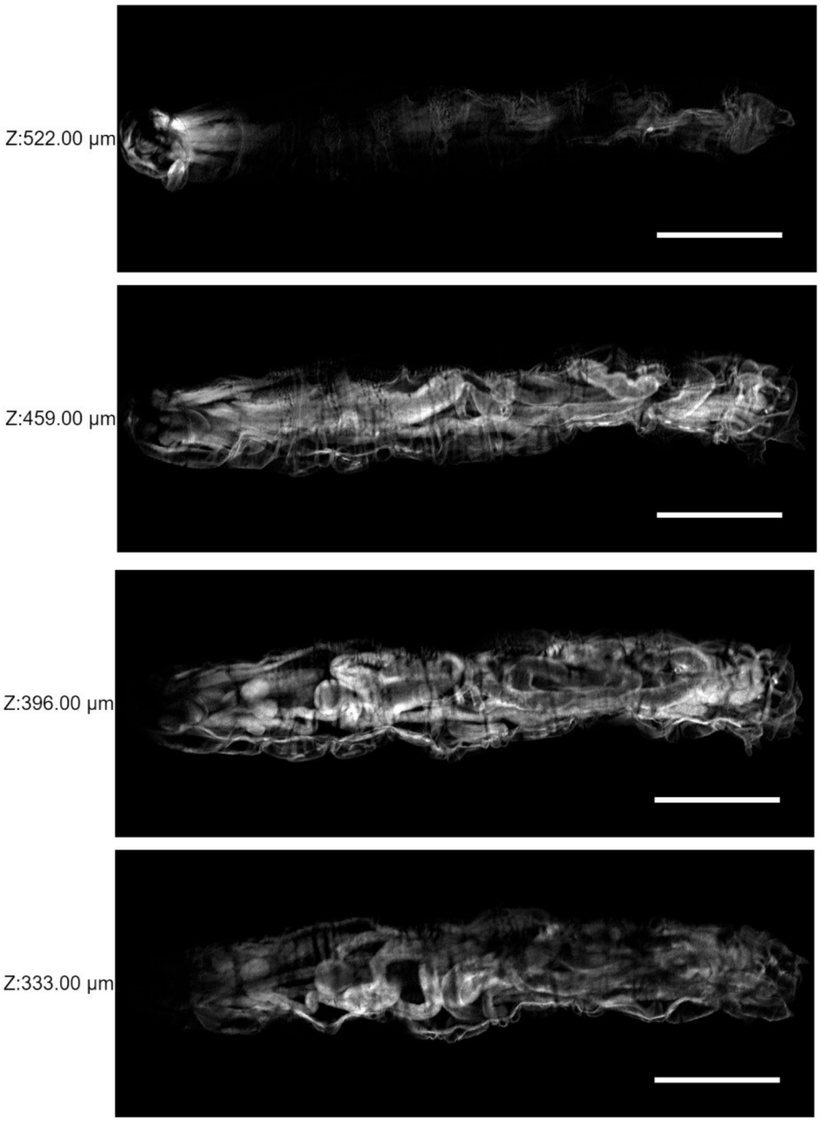

Figure S4

Fig. S4. Z-series of 3D autofluorescence imaging of *Drosophila melanogaster* larva. Scale bar 500 µm.

Biology Open • Supplementary information

Z:12
Z:11
Z:10
Z:9

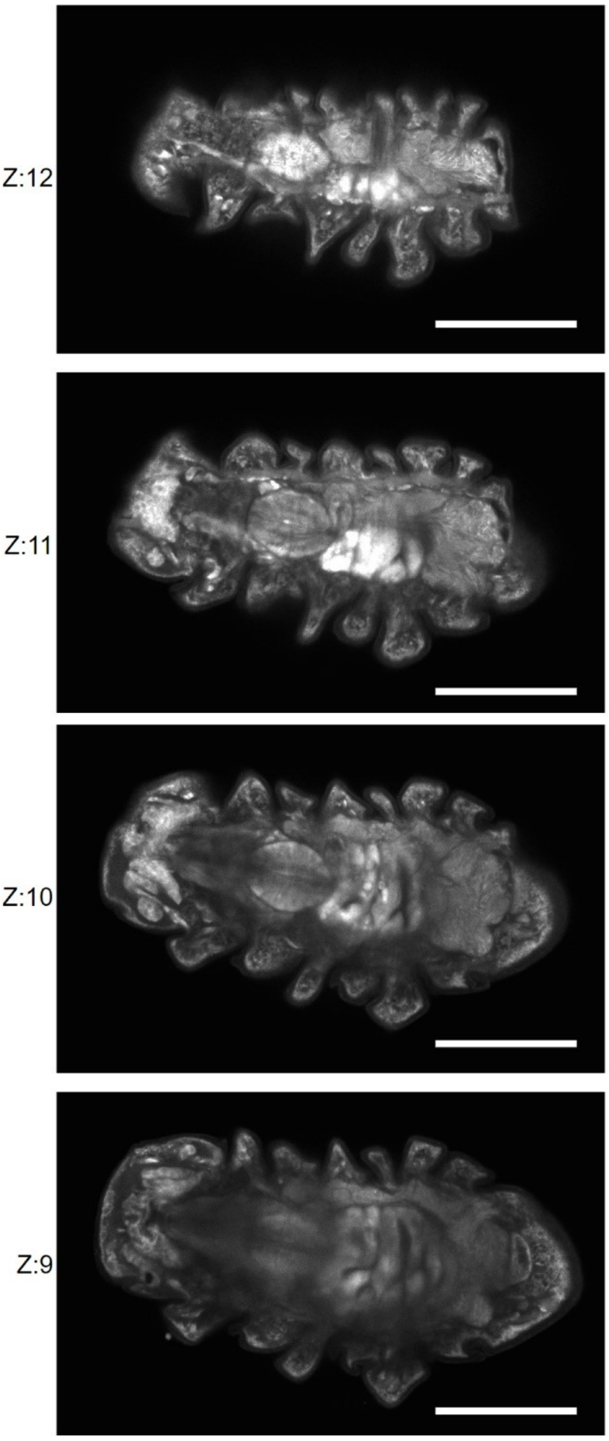

Figure S5

Fig. S5. Z-series of 3D autofluorescence imaging of tardigrade *Macrobiotus ripperi.* Scale bar 50 µm.

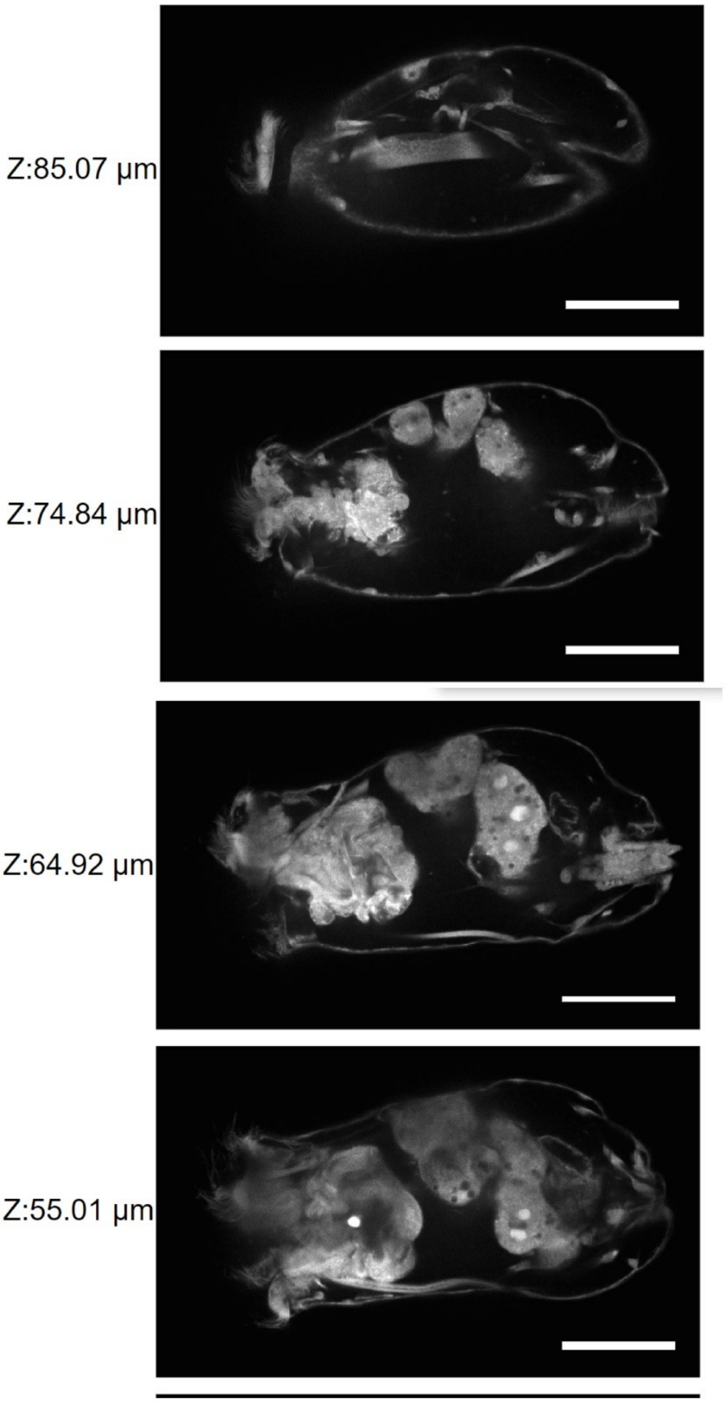

Figure S6

Fig. S6. Z-series of 3D autofluorescence imaging of rotifer *Brachionus plicatilis*. Scale bar 50 µm.

Biology Open • Supplementary information

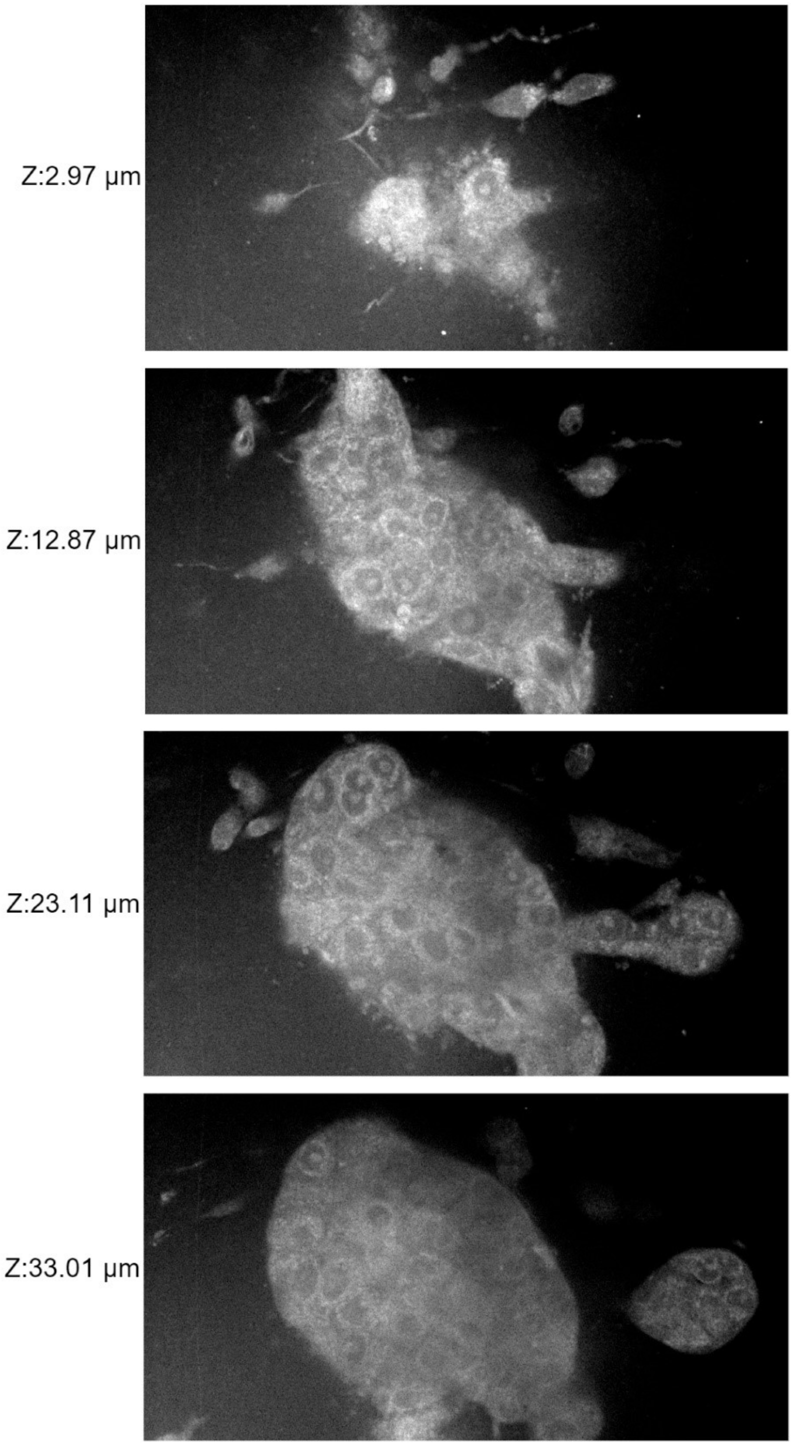

Figure S7

Fig. S7. Z-series of 3D autofluorescence imaging of human 3D-bioprinted mammary epithelial cell cultures .

Biology Open • Supplementary information

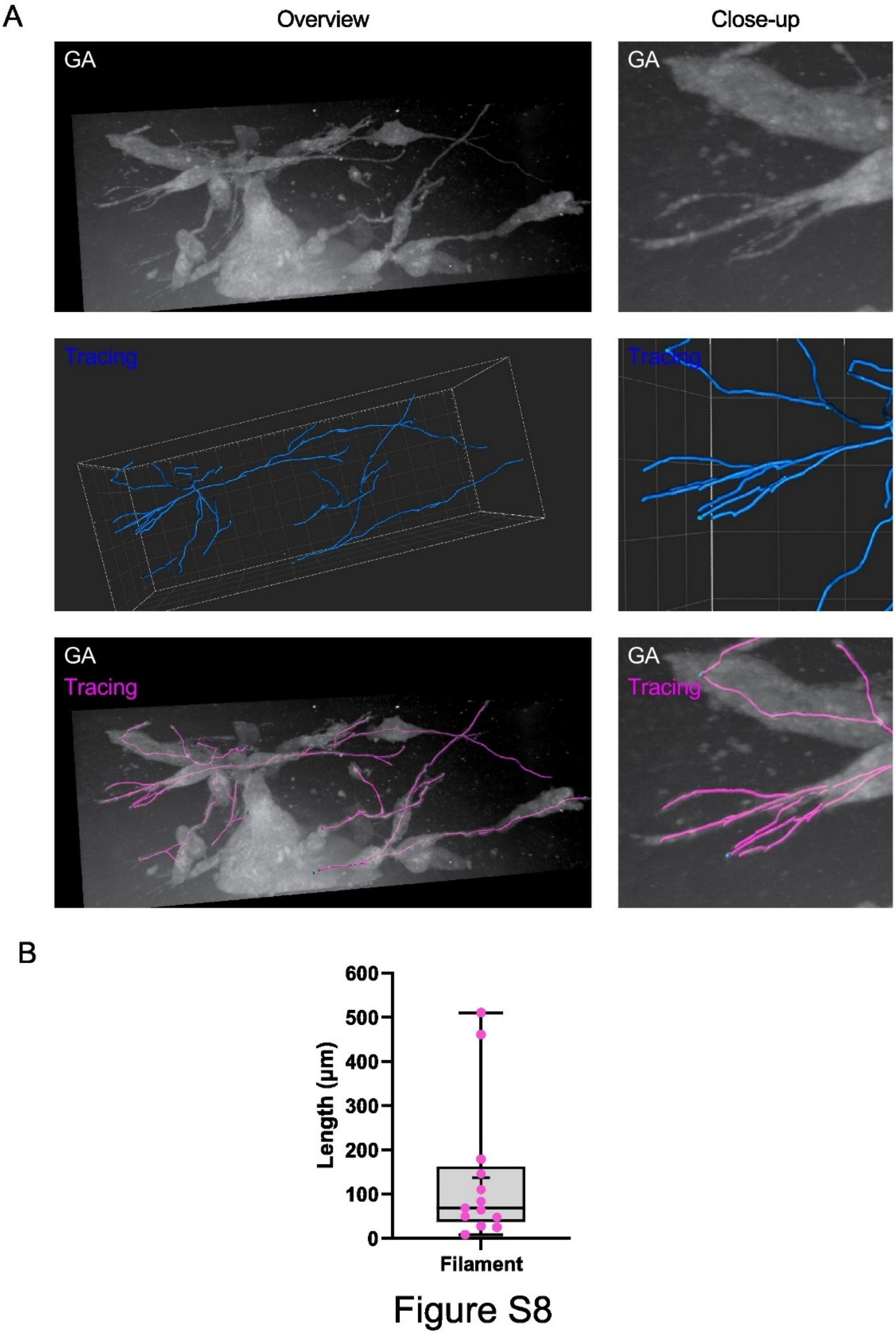

Fig. S8. Quantitative analyses of protrusions in human 3D-bioprinted mammary epithelial cell cultures. A) 3D view of Fluorescence and results of filament tracing using Imaris. B) Measurement of filament lengths in 3D presented as box plot. Mean marked as +, n= 13 filaments.

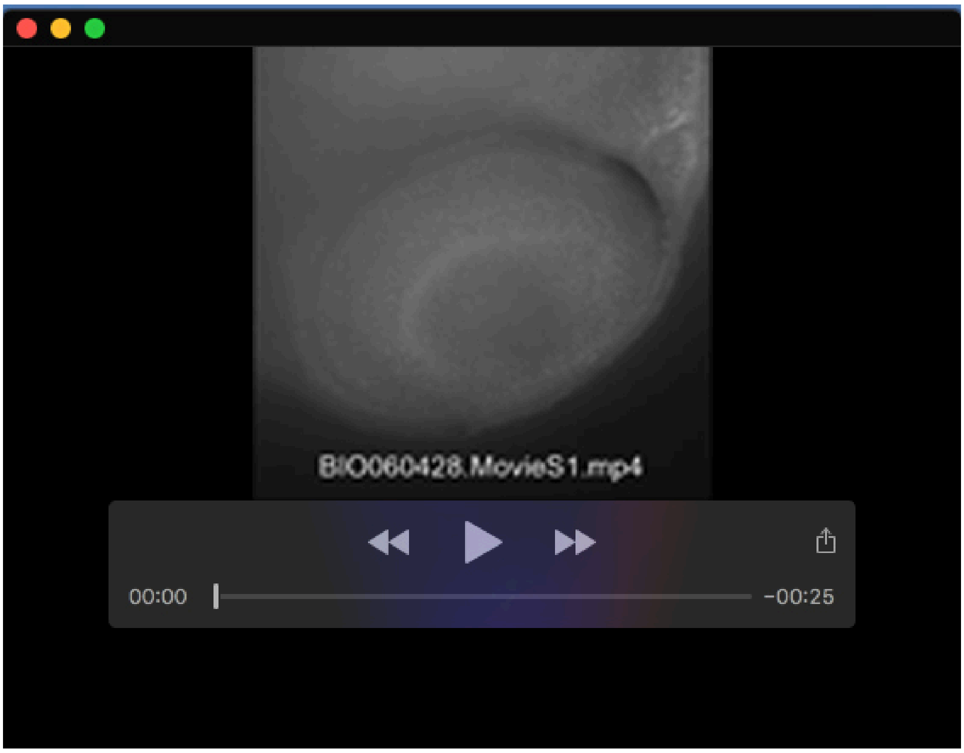

Movie 1. Scan through eye of whole-mount zebrafish embryo imaged with spinning disk confocal.

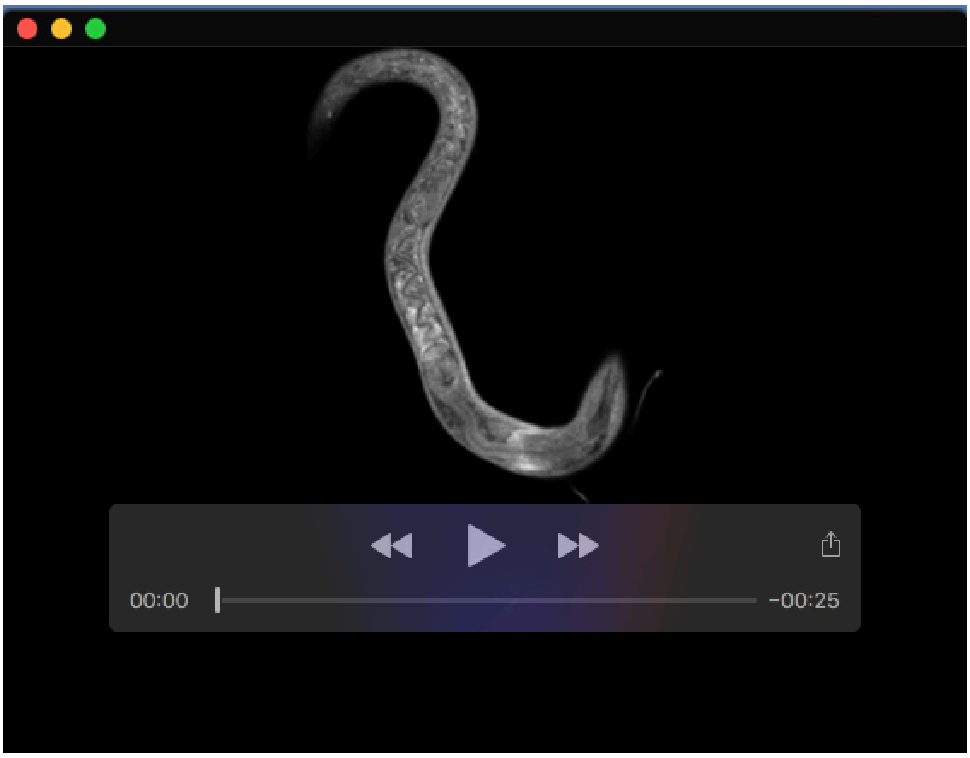

Movie 2. Scan through autofluorescence enhanced *Caenorhabditis elegans* imaged with spinning disk confocal.

Biology Open • Supplementary information

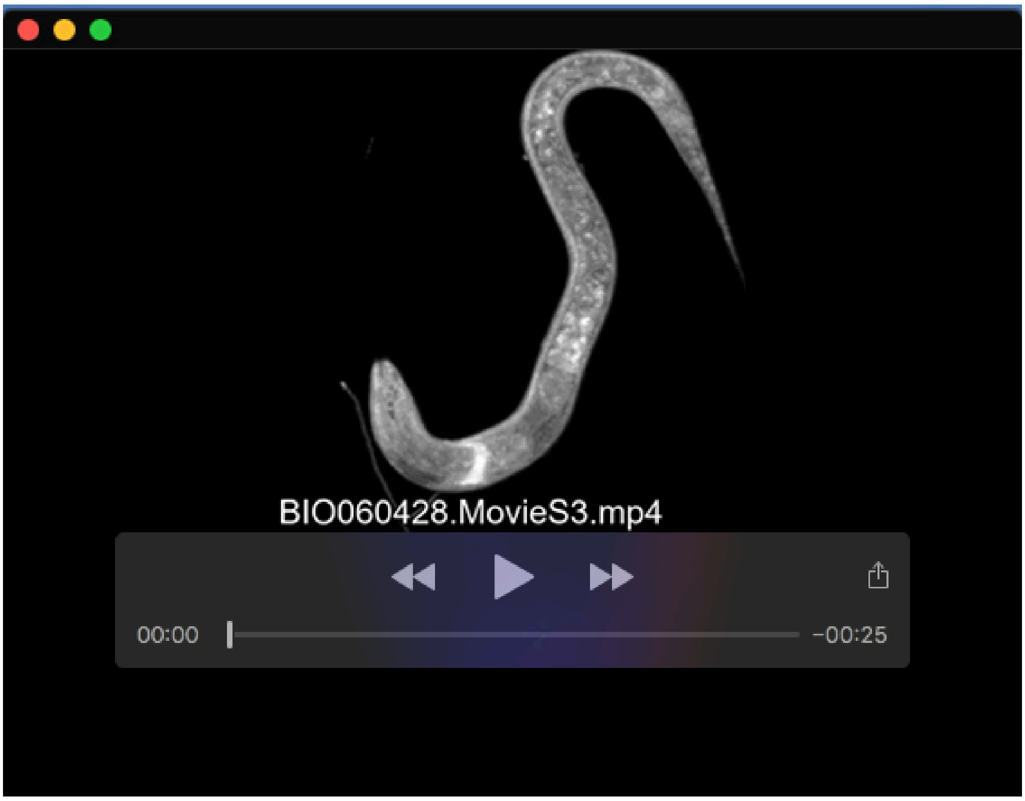

Movie 3. 3D rendering of *Caenorhabditis elegans*.

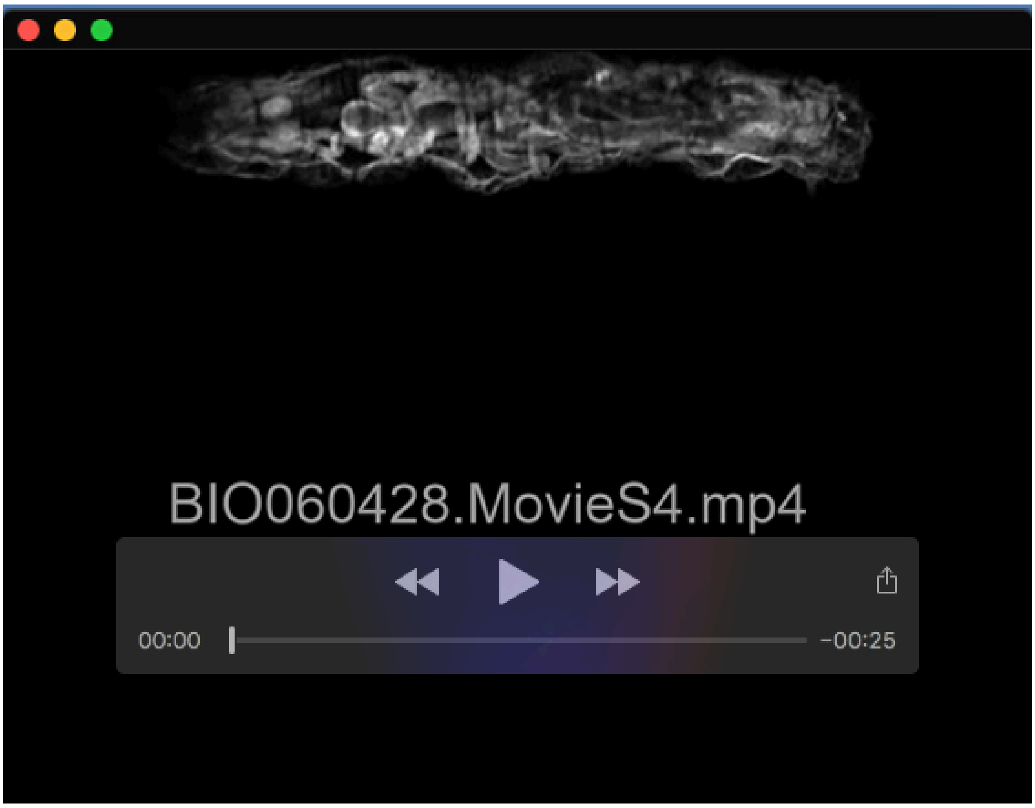

Movie 4. Scan through whole-mount *Drosophila melanogaster* larva.

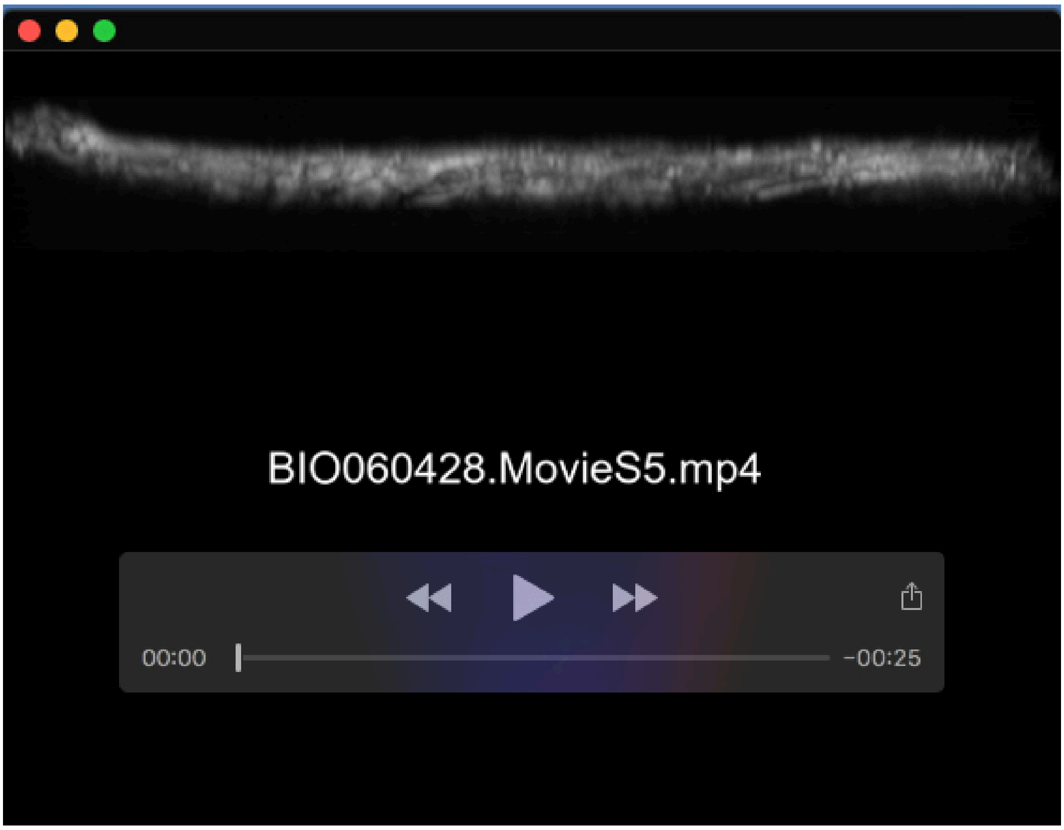

Movie 5. 3D rendering of whole-mount *Drosophila melanogaster* larva.

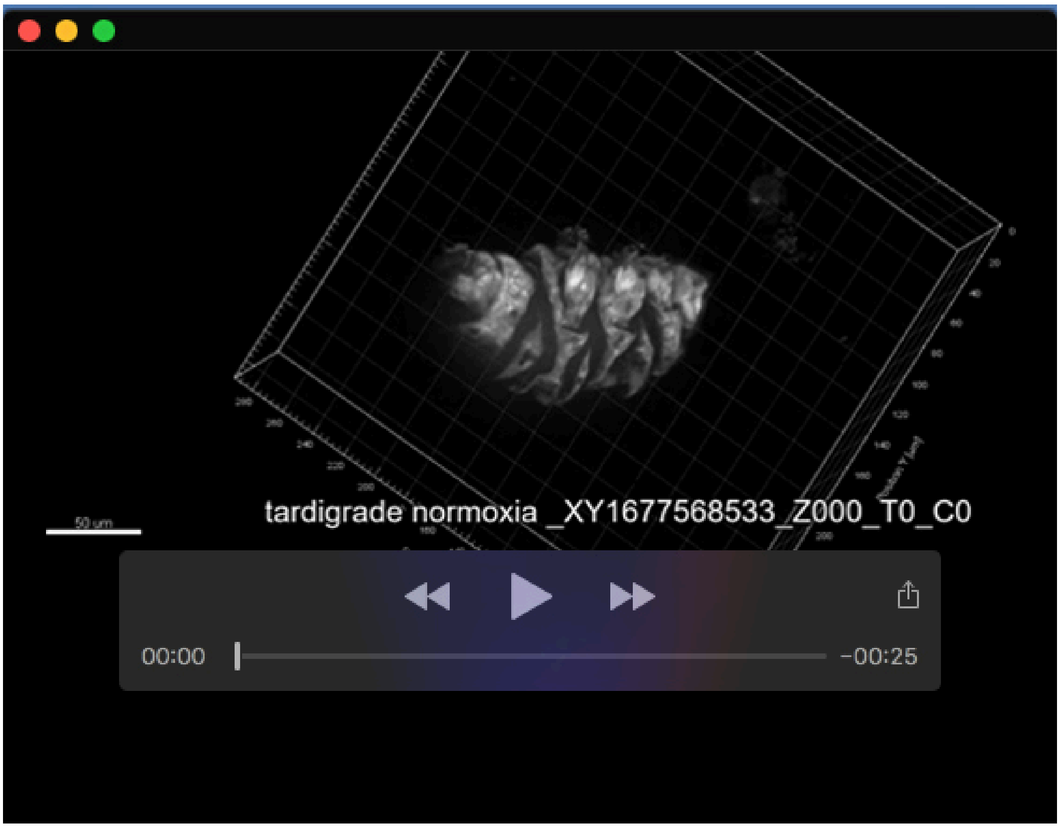

Movie 6. 3D rendering of whole-mount tardigrade *Macrobiotus ripperi*.

Biology Open • Supplementary information

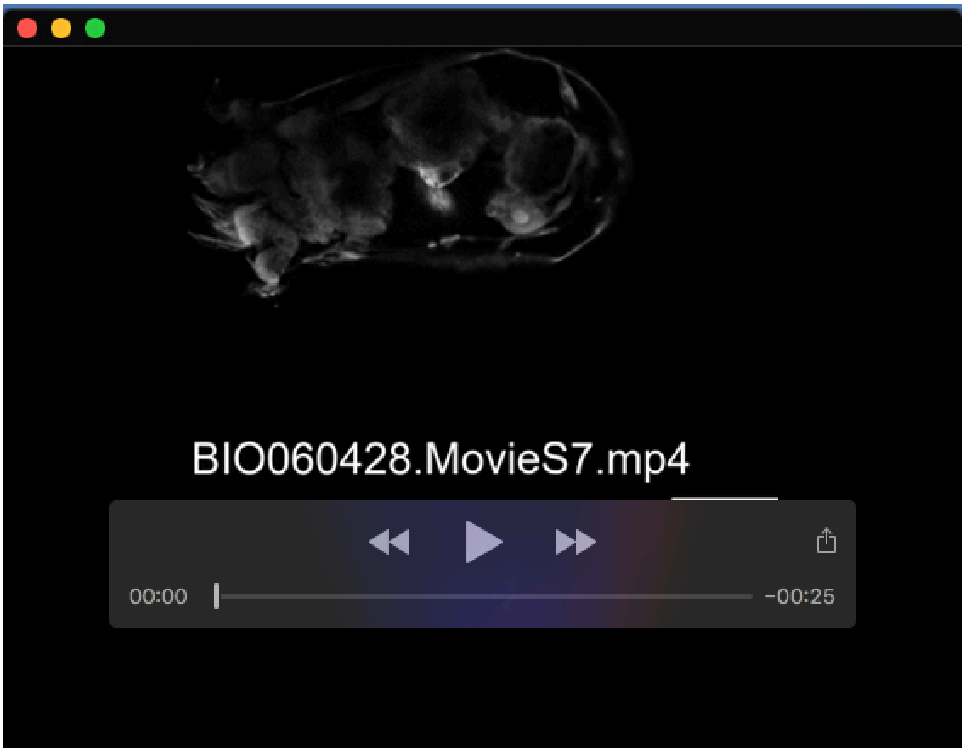

Movie 7. Scan through whole-mount rotifer *Brachionus plicatilis* processed with autofluorescence enhancement and imaged with spinning disk confocal.

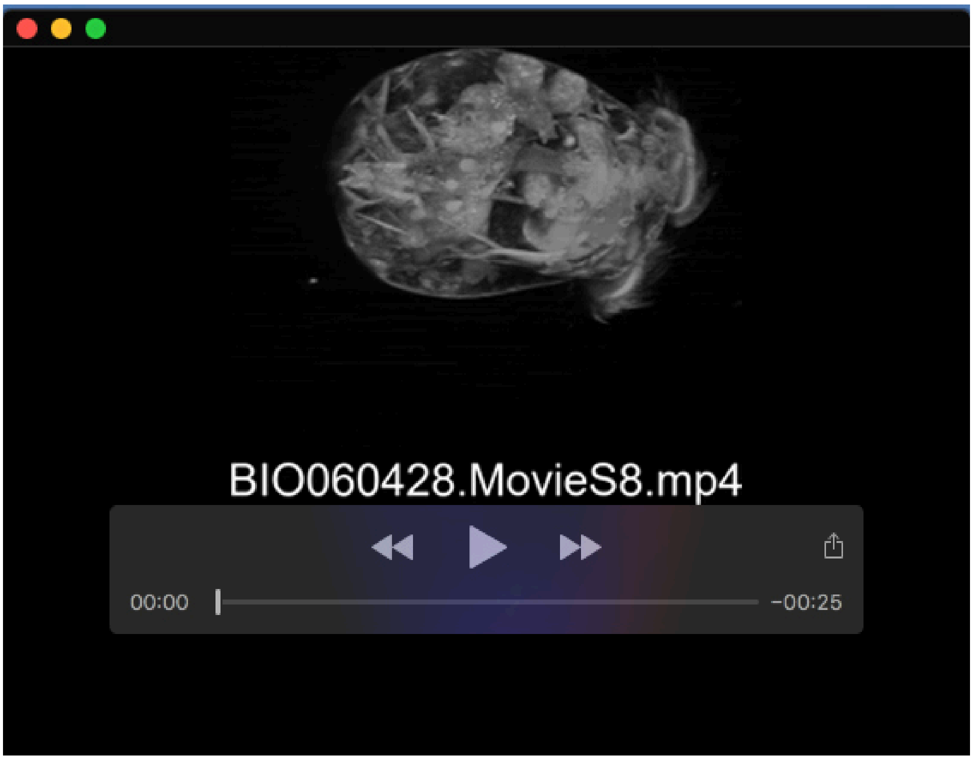

Movie 8. 3D rendering of whole-mount rotifer *Brachionus plicatilis* processed with autofluorescence enhancement and imaged with spinning disk confocal.

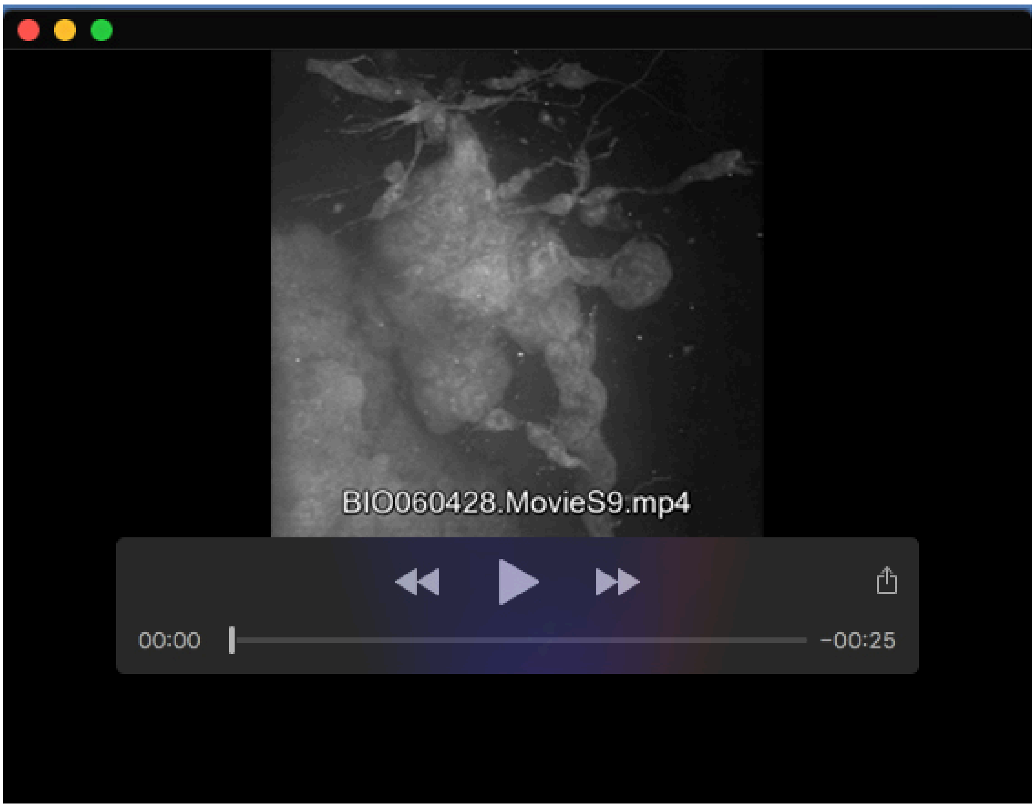

Movie 9. 3D rendering of human 3D-bioprinted mammary epithelial cell culture.
